# Di-μ-chlorido-bis­{[μ-1,8-bis­(diisopropyl­phosphan­yl)-9,10-dihydro-9,10-ethano­anthracene-κ^2^ *P*:*P*′]-μ-chlorido-μ-methyl­idene-dipalladium(II)} tetra­hydro­furan penta­solvate

**DOI:** 10.1107/S1600536810010263

**Published:** 2010-03-27

**Authors:** Thomas Schnetz, Frank Rominger, Peter Hofmann

**Affiliations:** a Organisch-Chemisches Institut, Universität Heidelberg, Im Neuenheimer Feld 270, 69120 Heidelberg, Germany

## Abstract

The title compound, [Pd_4_(CH_2_)_2_Cl_4_(C_28_H_40_P_2_)_2_]·5C_4_H_8_O, possesses a tetra­nuclear palladium core with four bridging chlorido ligands and two bridging methyl­ene units, as well as two bridging anthracene-based bis-phosphine ligands. This tetra­nuclear complex can be considered as being composed of two μ-chlorido-bridged *L*Pd_2_ units. The structural motif of these *L*Pd_2_ units shows two doubly bridged palladium centers between the P atoms of the bis-phosphine ligand. One of these bridges is a μ-Cl atom, the other a μ-methyl­ene group. The coordination environment around each palladium center is essentially square planar. We ascribe the oxidation state +II to the palladium centers and do not assume Pd—Pd bonds [shortest distances 2.8110 (5) and 2.8109 (6) Å]. Co-crystallized with the palladium complex we found five non-coordinating tetra­hydro­furan solvent mol­ecules, one of which is disordered over two positions in a 0.429 (9):0.571 (8) ratio.

## Related literature

For the synthesis of the Pd(0) precursor, see: Schnetz *et al.* (2008 ▶). The structural motif of a doubly brigded dinuclear Pd(II) complex with anthraceno-based bis-phosphines was reported by Warth (1999 ▶) and Grossman *et al.* (2006 ▶). Related palladium structures with a bridging μ_2_-methyl­ene group were reported by Brownie *et al.* (2003 ▶) and Klopfenstein *et al.* (1996 ▶). For a recent example of a tetra­nuclear Pd platform with bridging μ_2_-methyl­ene units, see: Sachse *et al.* (2010 ▶). 
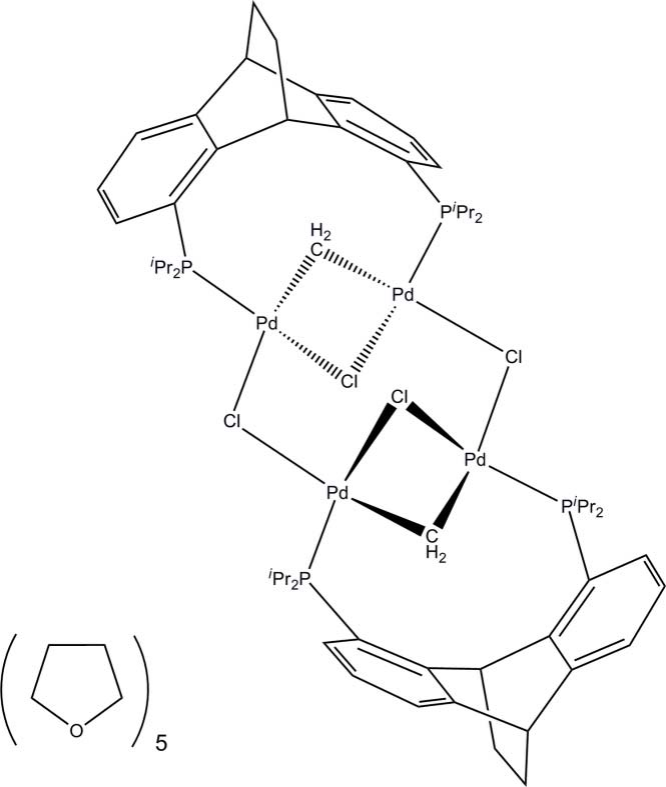


## Experimental

### 

#### Crystal data

[Pd_4_(CH_2_)_2_Cl_4_(C_28_H_40_P_2_)_2_]·5C_4_H_8_O
*M* *_r_* = 1833.05
Triclinic, 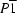

*a* = 12.5799 (1) Å
*b* = 17.2830 (3) Å
*c* = 19.7558 (2) Å
α = 79.877 (1)°
β = 74.703 (1)°
γ = 80.717 (1)°
*V* = 4048.61 (9) Å^3^
*Z* = 2
Mo *K*α radiation
μ = 1.13 mm^−1^
*T* = 200 K
0.18 × 0.18 × 0.06 mm

#### Data collection

Bruker SMART CCD diffractometer
Absorption correction: multi-scan (*SADABS*; Sheldrick, 2008*a* ▶) *T*_min_ = 0.822, *T*_max_ = 0.935
36611 measured reflections
15292 independent reflections
9483 reflections with *I* > 2σ(*I*)
*R*_int_ = 0.056

#### Refinement

*R*[*F* ^2^ > 2σ(*F* ^2^)] = 0.047
*wR*(*F* ^2^) = 0.106
*S* = 1.01
15292 reflections
915 parameters
434 restraints
H atoms treated by a mixture of independent and constrained refinement
Δρ_max_ = 0.82 e Å^−3^
Δρ_min_ = −0.73 e Å^−3^

### 

Data collection: *SMART* (Siemens, 1996 ▶); cell refinement: *SAINT* (Siemens, 1996 ▶); data reduction: *SAINT*; program(s) used to solve structure: *SHELXTL* (Sheldrick, 2008*b* ▶); program(s) used to refine structure: *SHELXTL*; molecular graphics: *SHELXTL*; software used to prepare material for publication: *SHELXTL*.

## Supplementary Material

Crystal structure: contains datablocks I, global. DOI: 10.1107/S1600536810010263/hg2653sup1.cif

Structure factors: contains datablocks I. DOI: 10.1107/S1600536810010263/hg2653Isup2.hkl

Additional supplementary materials:  crystallographic information; 3D view; checkCIF report

## Figures and Tables

**Fig. 1.**
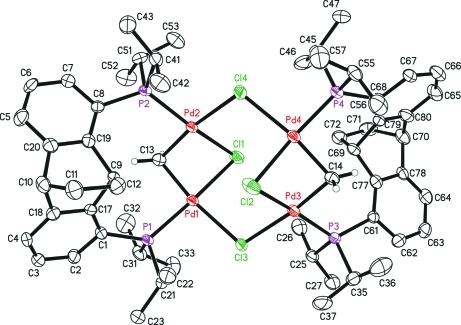
Thermal ellipsoid representation of the title compound. Displacement ellipsoids were plotted at 50% probability level.

## supplementary crystallographic information

### Comment

The tetranuclear complex can be considered as composed of two LPd_2_ units, bridged by two µ-chloro units. The structural motif of these LPd_2_ units with two twofold bridged metal centers between the phosphorus atoms of the chelating ligand is known in our group (Warth, 1999) and others (Grossman *et al.*, 2006). The anthraceno-based bis-phosphine ligand family shows a remarkable flexibility with respect to the P—P distances. Distances from 3.7 Å to 5.9 Å were found in the course of our investigations (5.607 (2) and 5.638 (2) in the title compound). This results in an excellent stabilization of different coordination numbers (2-6), geometries (trigonal-planar, cis- and trans-square planar, e,a- and e,e-trigonal-bipyramidal, octahedral) as well as dinuclear species. The coordination environment around each palladium center is essentially square planar, and we do not assume a Pd—Pd bond (Pd—Pd distance 2.8110 (5) Å resp. 2.8109 (6) Å). Structures with a bridging methylene group between palladium centers are very rare (Brownie *et al.*, 2003; Klopfenstein *et al.*, 1996; Sachse *et al.*, 2010). The palladium-carbon distances in the title compound (Pd—C 2.004 (5) Å to 2.017 (5) Å) are in good agreement with the values previously found, the Pd—C—Pd angles however (Pd—C—Pd 88.4 (2)° and 88.7 (2)°) are controlled by the otherwise determined Pd—Pd distance. In the hitherto known cases the angles spread over a wide range from 84.8 (2)° (Brownie *et al.*, 2003) to 109.4 (5)° (Sachse *et al.*, 2010).

### Experimental

In a reaction of [1,8-Bis(di-*iso*-propylphosphino)-9,10-dihydro-9,10-ethanoanthracene- κ^2^*P*,*P*']palladium(0) with an excess of n-Propylchloride in d_8_-THF at room temperature colourless crystals of the title compound could be obtained in small quantities under an inert argon atmosphere.

### Refinement

Five THF solvent molecules were located in the asymmetric unit, one of them had to be modelled disordered over two different orientations (occupancies 0.429 (8) : 571 (8)). All the solvent positions were refined using restraints keeping chemically equivalent bonds and angles similar, within one molecule as well as among the molecules. Rigid bond restraints and similarity restraints for spatially adjacent atoms were applied to the U_ij_ values of the solvent atoms. While the oxygen atoms of the four ordered THF molecules could be clearly identified, the assignments for the disordered THF molecule are not without ambiguity.

Hydrogen atoms of the phosphine ligands and the THF molecules were placed in calculated positions (C–H 0.95– 1.00 Å) and were included in the refinement in the riding model approximation with *U*_iso_(H) set to 1.2–1.5 *U*_eq_(C). A staggered group model was used for the methyl groups. The hydrogen atoms of the bridging methylene groups could be clearly identified in the difference electron density map and were treated in the refinement using restraints for similar C–H distances and a common displacement parameter.

### Crystal data

**Table:**

[Pd_4_(CH_2_)_2_Cl_4_(C_28_H_40_P_2_)_2_]·5C_4_H_8_O	*V* = 4048.61 (9) Å^3^
*M**_r_* = 1833.05	*Z* = 2
Triclinic, *P*1	*F*(000) = 1888
Hall symbol: -P 1	*D*_x_ = 1.504 Mg m^−^^3^
*a* = 12.5799 (1) Å	Mo *K*α radiation, λ = 0.71073 Å
*b* = 17.2830 (3) Å	Cell parameters from 5889 reflections
*c* = 19.7558 (2) Å	µ = 1.13 mm^−^^1^
α = 79.877 (1)°	*T* = 200 K
β = 74.703 (1)°	Polyhedron, colourless
γ = 80.717 (1)°	0.18 × 0.18 × 0.06 mm

### Data collection

**Table:**

Bruker SMART CCD diffractometer	15292 independent reflections
Radiation source: fine-focus sealed tube	9483 reflections with *I* > 2σ(*I*)
graphite	*R*_int_ = 0.056
ω scans	θ_max_ = 25.7°, θ_min_ = 1.7°
Absorption correction: multi-scan (*SADABS*; Sheldrick, 2008a)	*h* = −15→15
*T*_min_ = 0.822, *T*_max_ = 0.935	*k* = −21→21
36611 measured reflections	*l* = −24→24

### Refinement

**Table:**

Refinement on *F*^2^	Primary atom site location: structure-invariant direct methods
Least-squares matrix: full	Secondary atom site location: difference Fourier map
*R*[*F*^2^ > 2σ(*F*^2^)] = 0.047	Hydrogen site location: inferred from neighbouring sites
*wR*(*F*^2^) = 0.106	H atoms treated by a mixture of independent and constrained refinement
*S* = 1.01	*w* = 1/[σ^2^(*F*_o_^2^) + (0.0417*P*)^2^ + 0.8053*P*] where *P* = (*F*_o_^2^ + 2*F*_c_^2^)/3
15292 reflections	(Δ/σ)_max_ = 0.001
915 parameters	Δρ_max_ = 0.82 e Å^−^^3^
434 restraints	Δρ_min_ = −0.73 e Å^−^^3^

### Special details

**Table:**

Geometry. All esds (except the esd in the dihedral angle between two l.s. planes) are estimated using the full covariance matrix. The cell esds are taken into account individually in the estimation of esds in distances, angles and torsion angles; correlations between esds in cell parameters are only used when they are defined by crystal symmetry. An approximate (isotropic) treatment of cell esds is used for estimating esds involving l.s. planes.
Refinement. Refinement of *F*^2^ against ALL reflections. The weighted *R*-factor wR and goodness of fit *S* are based on *F*^2^, conventional *R*-factors *R* are based on *F*, with *F* set to zero for negative *F*^2^. The threshold expression of *F*^2^ > σ(*F*^2^) is used only for calculating *R*-factors(gt) etc. and is not relevant to the choice of reflections for refinement. *R*-factors based on *F*^2^ are statistically about twice as large as those based on *F*, and *R*- factors based on ALL data will be even larger.

### Fractional atomic coordinates and isotropic or equivalent isotropic displacement parameters (Å^2^)

**Table:**

	*x*	*y*	*z*	*U*_iso_*/*U*_eq_	Occ. (<1)
Pd1	0.47930 (3)	0.21727 (2)	0.34085 (2)	0.02319 (11)	
Pd2	0.24625 (3)	0.22887 (3)	0.38000 (2)	0.02516 (11)	
Pd3	0.47029 (3)	0.30751 (2)	0.14456 (2)	0.02341 (11)	
Pd4	0.23706 (3)	0.32848 (3)	0.18531 (2)	0.02701 (12)	
Cl1	0.36596 (11)	0.16459 (9)	0.28118 (7)	0.0361 (4)	
Cl2	0.35972 (11)	0.38581 (9)	0.23512 (8)	0.0430 (4)	
Cl3	0.59914 (10)	0.25954 (9)	0.22297 (7)	0.0339 (4)	
Cl4	0.11979 (11)	0.29472 (11)	0.30539 (7)	0.0504 (5)	
C13	0.3675 (4)	0.1783 (3)	0.4288 (3)	0.0288 (13)	
H13A	0.373 (4)	0.1221 (15)	0.430 (2)	0.020 (6)*	
H13B	0.368 (4)	0.190 (3)	0.4733 (16)	0.020 (6)*	
C14	0.3510 (4)	0.3541 (3)	0.0940 (3)	0.0251 (13)	
H14A	0.344 (4)	0.332 (2)	0.0546 (18)	0.020 (6)*	
H14B	0.357 (4)	0.4093 (15)	0.085 (2)	0.020 (6)*	
P1	0.59170 (11)	0.24153 (8)	0.40365 (7)	0.0233 (3)	
P2	0.12809 (11)	0.25216 (8)	0.48211 (7)	0.0234 (3)	
P3	0.58086 (11)	0.25881 (8)	0.04890 (7)	0.0220 (3)	
P4	0.11326 (11)	0.30836 (9)	0.12971 (7)	0.0258 (3)	
C1	0.5481 (4)	0.2807 (3)	0.4894 (3)	0.0237 (12)	
C2	0.6275 (4)	0.2669 (3)	0.5296 (3)	0.0298 (13)	
H2	0.6915	0.2301	0.5163	0.036*	
C3	0.6167 (5)	0.3044 (4)	0.5876 (3)	0.0357 (15)	
H3	0.6724	0.2928	0.6136	0.043*	
C4	0.5254 (4)	0.3586 (3)	0.6083 (3)	0.0284 (13)	
H4	0.5192	0.3869	0.6465	0.034*	
C5	0.1723 (5)	0.3546 (3)	0.6725 (3)	0.0369 (15)	
H5	0.1714	0.3818	0.7105	0.044*	
C6	0.0989 (4)	0.2990 (4)	0.6812 (3)	0.0359 (15)	
H6	0.0515	0.2847	0.7264	0.043*	
C7	0.0959 (4)	0.2652 (3)	0.6240 (3)	0.0319 (14)	
H7	0.0437	0.2289	0.6304	0.038*	
C8	0.1666 (4)	0.2818 (3)	0.5564 (3)	0.0241 (12)	
C9	0.3453 (4)	0.3529 (3)	0.4850 (3)	0.0210 (12)	
H9	0.3491	0.3241	0.4446	0.025*	
C10	0.3352 (4)	0.4230 (3)	0.5926 (3)	0.0294 (13)	
H10	0.3321	0.4480	0.6351	0.035*	
C11	0.3217 (5)	0.4861 (3)	0.5282 (3)	0.0371 (15)	
H11A	0.2486	0.5186	0.5395	0.045*	
H11B	0.3803	0.5215	0.5169	0.045*	
C12	0.3306 (4)	0.4437 (3)	0.4638 (3)	0.0308 (13)	
H12A	0.2627	0.4599	0.4460	0.037*	
H12B	0.3948	0.4595	0.4252	0.037*	
C17	0.4513 (4)	0.3315 (3)	0.5133 (3)	0.0217 (12)	
C18	0.4433 (4)	0.3705 (3)	0.5715 (3)	0.0274 (13)	
C19	0.2483 (4)	0.3315 (3)	0.5492 (3)	0.0230 (12)	
C20	0.2466 (4)	0.3694 (3)	0.6072 (3)	0.0275 (13)	
C21	0.6853 (4)	0.3135 (3)	0.3499 (3)	0.0307 (14)	
H21	0.7142	0.2958	0.3022	0.037*	
C22	0.6176 (5)	0.3940 (3)	0.3385 (3)	0.0430 (16)	
H22A	0.5531	0.3879	0.3217	0.064*	
H22B	0.6638	0.4299	0.3031	0.064*	
H22C	0.5922	0.4161	0.3833	0.064*	
C23	0.7874 (5)	0.3200 (4)	0.3761 (3)	0.0456 (17)	
H23A	0.8286	0.2673	0.3835	0.068*	
H23B	0.7636	0.3421	0.4209	0.068*	
H23C	0.8353	0.3547	0.3407	0.068*	
C25	0.6822 (4)	0.1773 (3)	0.0762 (3)	0.0274 (13)	
H25	0.7117	0.1968	0.1116	0.033*	
C26	0.6233 (5)	0.1063 (3)	0.1158 (3)	0.0389 (15)	
H26A	0.5599	0.1235	0.1532	0.058*	
H26B	0.6751	0.0671	0.1370	0.058*	
H26C	0.5970	0.0824	0.0827	0.058*	
C27	0.7842 (4)	0.1526 (4)	0.0180 (3)	0.0414 (16)	
H27A	0.8208	0.1993	−0.0062	0.062*	
H27B	0.7606	0.1288	−0.0163	0.062*	
H27C	0.8363	0.1140	0.0395	0.062*	
C31	0.6835 (4)	0.1482 (3)	0.4194 (3)	0.0321 (14)	
H31	0.7397	0.1594	0.4429	0.038*	
C32	0.6169 (5)	0.0855 (3)	0.4689 (3)	0.0501 (18)	
H32A	0.5795	0.1054	0.5138	0.075*	
H32B	0.6670	0.0373	0.4780	0.075*	
H32C	0.5613	0.0735	0.4469	0.075*	
C33	0.7457 (5)	0.1193 (4)	0.3483 (3)	0.0468 (17)	
H33A	0.7885	0.1607	0.3181	0.070*	
H33B	0.6920	0.1077	0.3246	0.070*	
H33C	0.7962	0.0713	0.3571	0.070*	
C35	0.6651 (5)	0.3376 (3)	−0.0035 (3)	0.0330 (14)	
H35	0.7242	0.3138	−0.0415	0.040*	
C36	0.5920 (5)	0.4037 (3)	−0.0393 (3)	0.0535 (18)	
H36A	0.5580	0.3813	−0.0695	0.080*	
H36B	0.6379	0.4440	−0.0683	0.080*	
H36C	0.5337	0.4281	−0.0029	0.080*	
C37	0.7210 (5)	0.3691 (4)	0.0435 (3)	0.056 (2)	
H37A	0.7664	0.3255	0.0654	0.084*	
H37B	0.6643	0.3937	0.0806	0.084*	
H37C	0.7684	0.4087	0.0149	0.084*	
C41	0.0125 (4)	0.3310 (3)	0.4693 (3)	0.0301 (13)	
H41	−0.0166	0.3177	0.4309	0.036*	
C42	0.0557 (5)	0.4103 (3)	0.4422 (3)	0.0423 (16)	
H42A	0.1180	0.4054	0.4007	0.063*	
H42B	0.0807	0.4276	0.4795	0.063*	
H42C	−0.0037	0.4494	0.4291	0.063*	
C43	−0.0856 (5)	0.3370 (4)	0.5334 (3)	0.0494 (18)	
H43A	−0.1126	0.2852	0.5498	0.074*	
H43B	−0.1452	0.3758	0.5202	0.074*	
H43C	−0.0621	0.3538	0.5715	0.074*	
C45	0.0110 (4)	0.2437 (3)	0.1876 (3)	0.0341 (14)	
H45	−0.0166	0.2655	0.2336	0.041*	
C46	0.0685 (5)	0.1603 (4)	0.2042 (3)	0.0510 (18)	
H46A	0.1336	0.1633	0.2216	0.077*	
H46B	0.0920	0.1350	0.1611	0.077*	
H46C	0.0168	0.1291	0.2405	0.077*	
C47	−0.0916 (5)	0.2408 (4)	0.1605 (3)	0.0503 (18)	
H47A	−0.1279	0.2947	0.1506	0.075*	
H47B	−0.1435	0.2094	0.1965	0.075*	
H47C	−0.0689	0.2163	0.1169	0.075*	
C51	0.0572 (4)	0.1624 (3)	0.5190 (3)	0.0297 (13)	
H51	−0.0046	0.1748	0.5607	0.036*	
C52	0.1377 (5)	0.0938 (3)	0.5439 (3)	0.0471 (17)	
H52A	0.1682	0.1094	0.5796	0.071*	
H52B	0.1983	0.0804	0.5034	0.071*	
H52C	0.0981	0.0475	0.5647	0.071*	
C53	0.0074 (5)	0.1400 (4)	0.4633 (3)	0.0445 (16)	
H53A	−0.0442	0.1846	0.4484	0.067*	
H53B	−0.0325	0.0938	0.4836	0.067*	
H53C	0.0670	0.1272	0.4221	0.067*	
C55	0.0288 (4)	0.4056 (3)	0.1131 (3)	0.0326 (14)	
H55	−0.0322	0.3968	0.0926	0.039*	
C56	0.0986 (5)	0.4633 (3)	0.0601 (3)	0.0433 (16)	
H56A	0.1310	0.4407	0.0159	0.065*	
H56B	0.1582	0.4736	0.0795	0.065*	
H56C	0.0519	0.5131	0.0504	0.065*	
C57	−0.0237 (5)	0.4382 (4)	0.1828 (3)	0.0507 (18)	
H57A	−0.0688	0.3998	0.2157	0.076*	
H57B	−0.0709	0.4880	0.1740	0.076*	
H57C	0.0348	0.4478	0.2035	0.076*	
C61	0.5362 (4)	0.2169 (3)	−0.0176 (3)	0.0208 (12)	
C62	0.6145 (5)	0.2129 (3)	−0.0826 (3)	0.0335 (14)	
H62	0.6778	0.2402	−0.0922	0.040*	
C63	0.6036 (5)	0.1707 (3)	−0.1339 (3)	0.0371 (15)	
H63	0.6582	0.1697	−0.1775	0.045*	
C64	0.5121 (4)	0.1301 (3)	−0.1204 (3)	0.0316 (14)	
H64	0.5044	0.0994	−0.1539	0.038*	
C65	0.1548 (5)	0.1827 (4)	−0.0661 (3)	0.0404 (16)	
H65	0.1552	0.1531	−0.1024	0.049*	
C66	0.0753 (5)	0.2472 (4)	−0.0516 (3)	0.0433 (16)	
H66	0.0237	0.2641	−0.0803	0.052*	
C67	0.0705 (4)	0.2866 (3)	0.0037 (3)	0.0318 (14)	
H67	0.0136	0.3293	0.0136	0.038*	
C68	0.1480 (4)	0.2657 (3)	0.0465 (3)	0.0247 (12)	
C69	0.3374 (4)	0.1758 (3)	0.0579 (3)	0.0239 (12)	
H69	0.3418	0.2077	0.0945	0.029*	
C70	0.3274 (5)	0.0963 (3)	−0.0395 (3)	0.0360 (15)	
H70	0.3228	0.0681	−0.0784	0.043*	
C71	0.3235 (5)	0.0396 (3)	0.0305 (3)	0.0395 (15)	
H71A	0.3852	−0.0039	0.0235	0.047*	
H71B	0.2527	0.0163	0.0456	0.047*	
C72	0.3337 (5)	0.0862 (3)	0.0879 (3)	0.0335 (14)	
H72A	0.4023	0.0646	0.1039	0.040*	
H72B	0.2696	0.0800	0.1293	0.040*	
C77	0.4402 (4)	0.1786 (3)	−0.0054 (3)	0.0240 (12)	
C78	0.4326 (4)	0.1349 (3)	−0.0579 (3)	0.0255 (12)	
C79	0.2347 (4)	0.2052 (3)	0.0281 (3)	0.0252 (13)	
C80	0.2345 (4)	0.1623 (3)	−0.0263 (3)	0.0326 (14)	
O80	0.1835 (5)	0.6705 (3)	0.1947 (3)	0.0915 (19)	
C81	0.2354 (8)	0.6670 (5)	0.2510 (5)	0.095 (3)	
H81A	0.1924	0.7042	0.2840	0.114*	
H81B	0.3112	0.6824	0.2319	0.114*	
C82	0.2408 (6)	0.5849 (4)	0.2891 (4)	0.075 (2)	
H82A	0.2285	0.5845	0.3408	0.090*	
H82B	0.3130	0.5537	0.2712	0.090*	
C83	0.1470 (6)	0.5538 (4)	0.2719 (4)	0.066 (2)	
H83A	0.1613	0.4957	0.2721	0.080*	
H83B	0.0754	0.5669	0.3059	0.080*	
C84	0.1476 (7)	0.5960 (4)	0.1998 (4)	0.078 (3)	
H84A	0.1984	0.5653	0.1638	0.094*	
H84B	0.0721	0.6029	0.1916	0.094*	
O85	0.2286 (6)	−0.1162 (4)	0.3862 (3)	0.132 (3)	
C86	0.2504 (10)	−0.1239 (5)	0.3145 (4)	0.121 (4)	
H86A	0.1959	−0.1545	0.3064	0.146*	
H86B	0.3258	−0.1519	0.2985	0.146*	
C87	0.2416 (8)	−0.0422 (5)	0.2748 (4)	0.089 (3)	
H87A	0.3159	−0.0247	0.2536	0.106*	
H87B	0.2025	−0.0392	0.2368	0.106*	
C88	0.1768 (7)	0.0059 (4)	0.3300 (4)	0.080 (2)	
H88A	0.1943	0.0612	0.3186	0.096*	
H88B	0.0960	0.0057	0.3369	0.096*	
C89	0.2148 (8)	−0.0357 (5)	0.3923 (4)	0.096 (3)	
H89A	0.2859	−0.0187	0.3932	0.116*	
H89B	0.1591	−0.0246	0.4365	0.116*	
O90	0.0116 (9)	−0.1175 (5)	0.2291 (6)	0.174 (4)	
C91	0.0593 (9)	−0.1076 (9)	0.1565 (6)	0.184 (8)	
H91A	0.0489	−0.1535	0.1362	0.220*	
H91B	0.1401	−0.1061	0.1484	0.220*	
C92	0.0106 (9)	−0.0350 (7)	0.1205 (5)	0.120 (4)	
H92A	−0.0069	−0.0445	0.0768	0.144*	
H92B	0.0606	0.0068	0.1082	0.144*	
C93	−0.0921 (7)	−0.0134 (5)	0.1748 (5)	0.089 (3)	
H93A	−0.1055	0.0444	0.1756	0.107*	
H93B	−0.1575	−0.0307	0.1653	0.107*	
C94	−0.0704 (8)	−0.0555 (8)	0.2414 (5)	0.135 (4)	
H94A	−0.0475	−0.0187	0.2665	0.162*	
H94B	−0.1391	−0.0756	0.2720	0.162*	
O95	0.7300 (10)	0.5637 (7)	0.2061 (7)	0.079 (4)	0.429 (8)
C96	0.680 (2)	0.6400 (9)	0.1905 (16)	0.090 (6)	0.429 (8)
H96A	0.7356	0.6726	0.1579	0.107*	0.429 (8)
H96B	0.6503	0.6644	0.2345	0.107*	0.429 (8)
C97	0.591 (2)	0.6369 (12)	0.1573 (17)	0.112 (6)	0.429 (8)
H97A	0.5889	0.6804	0.1176	0.134*	0.429 (8)
H97B	0.5178	0.6400	0.1921	0.134*	0.429 (8)
C98	0.620 (2)	0.5577 (13)	0.1312 (16)	0.102 (7)	0.429 (8)
H98A	0.5544	0.5279	0.1460	0.122*	0.429 (8)
H98B	0.6445	0.5645	0.0789	0.122*	0.429 (8)
C99	0.7080 (17)	0.5161 (9)	0.1624 (11)	0.071 (5)	0.429 (8)
H99A	0.6856	0.4654	0.1905	0.085*	0.429 (8)
H99B	0.7754	0.5042	0.1248	0.085*	0.429 (8)
O95B	0.5847 (12)	0.5705 (7)	0.2323 (5)	0.133 (5)	0.571 (8)
C96B	0.612 (2)	0.6465 (9)	0.2023 (8)	0.102 (6)	0.571 (8)
H96C	0.6551	0.6655	0.2300	0.123*	0.571 (8)
H96D	0.5437	0.6840	0.2017	0.123*	0.571 (8)
C97B	0.678 (2)	0.6408 (10)	0.1300 (8)	0.114 (6)	0.571 (8)
H97C	0.7531	0.6558	0.1237	0.137*	0.571 (8)
H97D	0.6418	0.6758	0.0952	0.137*	0.571 (8)
C98B	0.6847 (17)	0.5567 (10)	0.1210 (9)	0.107 (6)	0.571 (8)
H98C	0.6732	0.5520	0.0743	0.129*	0.571 (8)
H98D	0.7575	0.5274	0.1255	0.129*	0.571 (8)
C99B	0.5943 (15)	0.5271 (9)	0.1789 (8)	0.090 (5)	0.571 (8)
H99C	0.5242	0.5348	0.1633	0.108*	0.571 (8)
H99D	0.6126	0.4700	0.1947	0.108*	0.571 (8)
O100	0.6310 (6)	−0.0810 (3)	0.2290 (3)	0.094 (2)	
C101	0.6399 (8)	−0.1579 (4)	0.2635 (4)	0.091 (3)	
H10A	0.7157	−0.1848	0.2467	0.109*	
H10B	0.5864	−0.1879	0.2535	0.109*	
C102	0.6167 (7)	−0.1553 (5)	0.3389 (4)	0.089 (3)	
H10C	0.6824	−0.1788	0.3574	0.107*	
H10D	0.5536	−0.1850	0.3647	0.107*	
C103	0.5893 (10)	−0.0716 (5)	0.3481 (4)	0.130 (4)	
H10E	0.5153	−0.0624	0.3808	0.156*	
H10F	0.6451	−0.0544	0.3675	0.156*	
C104	0.5895 (8)	−0.0291 (4)	0.2784 (4)	0.089 (3)	
H10G	0.5131	−0.0058	0.2762	0.107*	
H10H	0.6363	0.0145	0.2680	0.107*	

### Atomic displacement parameters (Å^2^)

**Table:**

	*U*^11^	*U*^22^	*U*^33^	*U*^12^	*U*^13^	*U*^23^
Pd1	0.0153 (2)	0.0337 (3)	0.0231 (2)	−0.00019 (18)	−0.00609 (18)	−0.01090 (19)
Pd2	0.0153 (2)	0.0391 (3)	0.0236 (2)	−0.00203 (19)	−0.00592 (18)	−0.0102 (2)
Pd3	0.0175 (2)	0.0314 (3)	0.0243 (2)	−0.00120 (18)	−0.00655 (18)	−0.01135 (19)
Pd4	0.0174 (2)	0.0401 (3)	0.0253 (3)	0.00263 (19)	−0.00767 (19)	−0.0111 (2)
Cl1	0.0238 (7)	0.0553 (10)	0.0362 (9)	−0.0054 (7)	−0.0060 (6)	−0.0261 (7)
Cl2	0.0267 (8)	0.0601 (11)	0.0519 (10)	0.0057 (7)	−0.0123 (7)	−0.0394 (8)
Cl3	0.0186 (7)	0.0640 (10)	0.0220 (8)	−0.0036 (7)	−0.0059 (6)	−0.0136 (7)
Cl4	0.0196 (8)	0.1063 (14)	0.0236 (8)	0.0052 (8)	−0.0084 (6)	−0.0112 (9)
C13	0.022 (3)	0.035 (3)	0.030 (3)	−0.003 (3)	−0.007 (3)	−0.006 (3)
C14	0.023 (3)	0.024 (3)	0.031 (3)	−0.002 (2)	−0.008 (3)	−0.009 (3)
P1	0.0143 (7)	0.0337 (9)	0.0235 (8)	−0.0005 (6)	−0.0064 (6)	−0.0081 (6)
P2	0.0138 (7)	0.0324 (9)	0.0249 (8)	0.0005 (6)	−0.0062 (6)	−0.0067 (6)
P3	0.0185 (7)	0.0271 (8)	0.0217 (8)	−0.0038 (6)	−0.0051 (6)	−0.0060 (6)
P4	0.0188 (7)	0.0350 (9)	0.0242 (8)	−0.0015 (6)	−0.0081 (6)	−0.0026 (7)
C1	0.024 (3)	0.028 (3)	0.021 (3)	−0.007 (2)	−0.008 (2)	−0.004 (2)
C2	0.022 (3)	0.043 (4)	0.024 (3)	0.003 (3)	−0.008 (2)	−0.006 (3)
C3	0.026 (3)	0.059 (4)	0.025 (3)	−0.011 (3)	−0.008 (3)	−0.006 (3)
C4	0.027 (3)	0.043 (4)	0.020 (3)	−0.010 (3)	−0.006 (2)	−0.012 (3)
C5	0.030 (3)	0.050 (4)	0.029 (4)	0.006 (3)	−0.002 (3)	−0.017 (3)
C6	0.025 (3)	0.058 (4)	0.020 (3)	−0.004 (3)	0.004 (2)	−0.006 (3)
C7	0.024 (3)	0.047 (4)	0.026 (3)	−0.008 (3)	−0.006 (3)	−0.005 (3)
C8	0.016 (3)	0.032 (3)	0.023 (3)	0.004 (2)	−0.005 (2)	−0.006 (2)
C9	0.014 (3)	0.030 (3)	0.020 (3)	0.000 (2)	−0.006 (2)	−0.005 (2)
C10	0.031 (3)	0.028 (3)	0.034 (3)	0.002 (3)	−0.011 (3)	−0.015 (3)
C11	0.043 (4)	0.031 (3)	0.040 (4)	−0.005 (3)	−0.011 (3)	−0.010 (3)
C12	0.030 (3)	0.031 (3)	0.028 (3)	0.000 (3)	−0.008 (3)	0.001 (3)
C17	0.016 (3)	0.029 (3)	0.020 (3)	−0.004 (2)	−0.005 (2)	−0.001 (2)
C18	0.028 (3)	0.031 (3)	0.023 (3)	−0.011 (3)	−0.004 (2)	0.000 (2)
C19	0.020 (3)	0.025 (3)	0.023 (3)	0.007 (2)	−0.009 (2)	−0.003 (2)
C20	0.021 (3)	0.030 (3)	0.032 (3)	0.005 (2)	−0.009 (3)	−0.010 (3)
C21	0.024 (3)	0.052 (4)	0.019 (3)	−0.014 (3)	−0.003 (2)	−0.006 (3)
C22	0.048 (4)	0.048 (4)	0.037 (4)	−0.021 (3)	−0.009 (3)	−0.006 (3)
C23	0.032 (4)	0.080 (5)	0.032 (4)	−0.022 (3)	−0.005 (3)	−0.014 (3)
C25	0.021 (3)	0.036 (3)	0.028 (3)	0.000 (2)	−0.007 (2)	−0.010 (3)
C26	0.038 (4)	0.036 (4)	0.043 (4)	0.008 (3)	−0.016 (3)	−0.007 (3)
C27	0.024 (3)	0.060 (4)	0.040 (4)	0.010 (3)	−0.008 (3)	−0.019 (3)
C31	0.025 (3)	0.038 (4)	0.037 (4)	0.008 (3)	−0.015 (3)	−0.012 (3)
C32	0.049 (4)	0.037 (4)	0.056 (5)	0.014 (3)	−0.015 (4)	−0.003 (3)
C33	0.042 (4)	0.053 (4)	0.043 (4)	0.022 (3)	−0.013 (3)	−0.021 (3)
C35	0.033 (3)	0.037 (4)	0.029 (3)	−0.013 (3)	−0.001 (3)	−0.007 (3)
C36	0.054 (4)	0.036 (4)	0.060 (5)	−0.010 (3)	−0.003 (4)	0.008 (3)
C37	0.054 (4)	0.075 (5)	0.048 (4)	−0.045 (4)	−0.002 (3)	−0.015 (4)
C41	0.026 (3)	0.038 (4)	0.027 (3)	0.002 (3)	−0.010 (3)	−0.008 (3)
C42	0.038 (4)	0.035 (4)	0.056 (4)	0.004 (3)	−0.023 (3)	−0.005 (3)
C43	0.031 (4)	0.076 (5)	0.037 (4)	0.022 (3)	−0.012 (3)	−0.018 (3)
C45	0.022 (3)	0.050 (4)	0.028 (3)	−0.008 (3)	−0.004 (3)	0.001 (3)
C46	0.036 (4)	0.054 (4)	0.053 (4)	−0.016 (3)	0.001 (3)	0.012 (3)
C47	0.026 (3)	0.081 (5)	0.045 (4)	−0.019 (3)	−0.008 (3)	0.000 (4)
C51	0.020 (3)	0.035 (3)	0.030 (3)	−0.005 (2)	0.001 (2)	−0.004 (3)
C52	0.045 (4)	0.032 (4)	0.063 (5)	−0.008 (3)	−0.016 (3)	0.003 (3)
C53	0.043 (4)	0.043 (4)	0.057 (4)	−0.013 (3)	−0.023 (3)	−0.010 (3)
C55	0.028 (3)	0.036 (4)	0.037 (4)	0.003 (3)	−0.018 (3)	−0.002 (3)
C56	0.048 (4)	0.034 (4)	0.050 (4)	−0.003 (3)	−0.022 (3)	0.004 (3)
C57	0.053 (4)	0.049 (4)	0.048 (4)	0.014 (3)	−0.015 (3)	−0.016 (3)
C61	0.018 (3)	0.023 (3)	0.021 (3)	0.001 (2)	−0.006 (2)	−0.004 (2)
C62	0.031 (3)	0.041 (4)	0.028 (3)	−0.011 (3)	−0.003 (3)	−0.005 (3)
C63	0.035 (4)	0.052 (4)	0.023 (3)	−0.002 (3)	−0.002 (3)	−0.012 (3)
C64	0.034 (3)	0.034 (3)	0.030 (3)	0.005 (3)	−0.011 (3)	−0.017 (3)
C65	0.043 (4)	0.050 (4)	0.040 (4)	−0.010 (3)	−0.020 (3)	−0.019 (3)
C66	0.031 (4)	0.063 (5)	0.043 (4)	−0.005 (3)	−0.020 (3)	−0.010 (3)
C67	0.020 (3)	0.048 (4)	0.028 (3)	0.000 (3)	−0.009 (3)	−0.007 (3)
C68	0.021 (3)	0.029 (3)	0.026 (3)	−0.006 (2)	−0.009 (2)	−0.003 (2)
C69	0.023 (3)	0.022 (3)	0.030 (3)	−0.005 (2)	−0.011 (2)	−0.004 (2)
C70	0.040 (4)	0.036 (4)	0.040 (4)	−0.004 (3)	−0.018 (3)	−0.016 (3)
C71	0.035 (4)	0.029 (3)	0.056 (4)	−0.011 (3)	−0.007 (3)	−0.009 (3)
C72	0.033 (3)	0.030 (3)	0.038 (4)	−0.008 (3)	−0.012 (3)	0.001 (3)
C77	0.026 (3)	0.018 (3)	0.028 (3)	0.002 (2)	−0.012 (2)	0.000 (2)
C78	0.026 (3)	0.030 (3)	0.024 (3)	−0.001 (2)	−0.011 (3)	−0.009 (2)
C79	0.023 (3)	0.034 (3)	0.023 (3)	−0.012 (3)	−0.007 (2)	−0.004 (2)
C80	0.029 (3)	0.034 (3)	0.040 (4)	−0.011 (3)	−0.013 (3)	−0.004 (3)
O80	0.132 (5)	0.070 (4)	0.068 (4)	−0.040 (4)	−0.005 (4)	−0.001 (3)
C81	0.107 (8)	0.091 (8)	0.086 (7)	−0.048 (6)	−0.003 (6)	−0.005 (6)
C82	0.078 (6)	0.053 (5)	0.083 (6)	−0.003 (4)	0.003 (5)	−0.025 (5)
C83	0.055 (5)	0.045 (5)	0.078 (6)	−0.002 (4)	0.016 (4)	−0.006 (4)
C84	0.087 (6)	0.076 (6)	0.066 (6)	−0.035 (5)	0.020 (5)	−0.030 (5)
O85	0.192 (8)	0.086 (5)	0.080 (5)	0.053 (5)	−0.016 (5)	0.000 (4)
C86	0.216 (13)	0.072 (7)	0.062 (6)	0.029 (7)	−0.023 (7)	−0.030 (5)
C87	0.125 (8)	0.086 (7)	0.050 (5)	0.005 (6)	−0.023 (5)	−0.010 (5)
C88	0.088 (7)	0.067 (6)	0.091 (7)	−0.003 (5)	−0.042 (6)	−0.008 (5)
C89	0.124 (9)	0.084 (7)	0.081 (7)	0.006 (6)	−0.035 (6)	−0.016 (6)
O90	0.161 (9)	0.121 (7)	0.222 (11)	−0.004 (6)	−0.038 (8)	−0.003 (7)
C91	0.090 (9)	0.36 (2)	0.109 (10)	0.087 (11)	−0.025 (8)	−0.158 (13)
C92	0.101 (9)	0.169 (12)	0.092 (8)	−0.061 (8)	0.008 (7)	−0.032 (8)
C93	0.072 (7)	0.078 (6)	0.125 (9)	−0.013 (5)	−0.036 (7)	−0.012 (6)
C94	0.070 (8)	0.192 (14)	0.117 (10)	0.002 (8)	0.009 (7)	−0.018 (10)
O95	0.079 (9)	0.079 (8)	0.098 (10)	0.016 (7)	−0.055 (8)	−0.037 (7)
C96	0.117 (15)	0.074 (9)	0.090 (15)	−0.010 (10)	−0.058 (12)	0.003 (10)
C97	0.142 (14)	0.099 (11)	0.117 (14)	0.035 (11)	−0.096 (11)	−0.023 (11)
C98	0.110 (16)	0.103 (13)	0.114 (16)	0.025 (12)	−0.081 (12)	−0.026 (12)
C99	0.093 (12)	0.077 (10)	0.052 (11)	0.005 (9)	−0.049 (9)	−0.004 (8)
O95B	0.203 (14)	0.092 (8)	0.075 (7)	−0.028 (8)	0.022 (8)	−0.010 (6)
C96B	0.162 (16)	0.072 (8)	0.075 (10)	−0.012 (10)	−0.027 (11)	−0.021 (7)
C97B	0.142 (14)	0.100 (10)	0.090 (11)	−0.063 (11)	0.010 (10)	0.001 (9)
C98B	0.124 (14)	0.107 (10)	0.079 (10)	0.020 (11)	−0.007 (9)	−0.037 (9)
C99B	0.137 (13)	0.071 (9)	0.082 (10)	−0.040 (9)	−0.062 (9)	0.017 (7)
O100	0.168 (6)	0.062 (4)	0.072 (4)	−0.023 (4)	−0.059 (4)	−0.006 (3)
C101	0.140 (9)	0.049 (6)	0.091 (7)	0.003 (5)	−0.048 (6)	−0.015 (5)
C102	0.105 (7)	0.086 (7)	0.071 (7)	−0.033 (6)	−0.020 (5)	0.024 (5)
C103	0.232 (14)	0.077 (7)	0.057 (7)	−0.033 (8)	0.010 (7)	−0.004 (5)
C104	0.128 (8)	0.053 (5)	0.086 (7)	0.011 (5)	−0.037 (6)	−0.011 (5)

### Geometric parameters (Å, °)

**Table:**

Pd1—C13	2.014 (6)	C52—H52B	0.9800
Pd1—P1	2.2393 (13)	C52—H52C	0.9800
Pd1—Cl1	2.4360 (13)	C53—H53A	0.9800
Pd1—Cl3	2.4767 (14)	C53—H53B	0.9800
Pd1—Pd2	2.8110 (5)	C53—H53C	0.9800
Pd2—C13	2.017 (5)	C55—C56	1.515 (7)
Pd2—P2	2.2251 (14)	C55—C57	1.524 (7)
Pd2—Cl1	2.4465 (14)	C55—H55	1.0000
Pd2—Cl4	2.4621 (14)	C56—H56A	0.9800
Pd3—C14	2.004 (5)	C56—H56B	0.9800
Pd3—P3	2.2380 (14)	C56—H56C	0.9800
Pd3—Cl2	2.4284 (14)	C57—H57A	0.9800
Pd3—Cl3	2.4807 (13)	C57—H57B	0.9800
Pd3—Pd4	2.8109 (6)	C57—H57C	0.9800
Pd4—C14	2.016 (5)	C61—C62	1.402 (7)
Pd4—P4	2.2281 (14)	C61—C77	1.416 (7)
Pd4—Cl2	2.4429 (14)	C62—C63	1.394 (7)
Pd4—Cl4	2.4676 (15)	C62—H62	0.9500
C13—H13A	0.96 (2)	C63—C64	1.386 (7)
C13—H13B	0.94 (2)	C63—H63	0.9500
C14—H14A	0.95 (2)	C64—C78	1.374 (7)
C14—H14B	0.95 (2)	C64—H64	0.9500
P1—C21	1.847 (5)	C65—C66	1.387 (8)
P1—C1	1.850 (5)	C65—C80	1.397 (7)
P1—C31	1.856 (5)	C65—H65	0.9500
P2—C8	1.836 (5)	C66—C67	1.370 (7)
P2—C51	1.854 (5)	C66—H66	0.9500
P2—C41	1.862 (5)	C67—C68	1.418 (7)
P3—C25	1.848 (5)	C67—H67	0.9500
P3—C61	1.850 (5)	C68—C79	1.405 (7)
P3—C35	1.854 (5)	C69—C79	1.536 (7)
P4—C68	1.838 (5)	C69—C77	1.544 (7)
P4—C45	1.857 (5)	C69—C72	1.562 (7)
P4—C55	1.864 (5)	C69—H69	1.0000
C1—C2	1.400 (7)	C70—C80	1.500 (8)
C1—C17	1.406 (7)	C70—C78	1.508 (7)
C2—C3	1.381 (7)	C70—C71	1.541 (8)
C2—H2	0.9500	C70—H70	1.0000
C3—C4	1.379 (7)	C71—C72	1.544 (7)
C3—H3	0.9500	C71—H71A	0.9900
C4—C18	1.382 (7)	C71—H71B	0.9900
C4—H4	0.9500	C72—H72A	0.9900
C5—C20	1.389 (7)	C72—H72B	0.9900
C5—C6	1.396 (8)	C77—C78	1.416 (7)
C5—H5	0.9500	C79—C80	1.408 (7)
C6—C7	1.372 (7)	O80—C84	1.410 (7)
C6—H6	0.9500	O80—C81	1.419 (8)
C7—C8	1.407 (7)	C81—C82	1.485 (8)
C7—H7	0.9500	C81—H81A	0.9900
C8—C19	1.407 (7)	C81—H81B	0.9900
C9—C17	1.545 (6)	C82—C83	1.509 (8)
C9—C12	1.545 (7)	C82—H82A	0.9900
C9—C19	1.549 (7)	C82—H82B	0.9900
C9—H9	1.0000	C83—C84	1.481 (8)
C10—C20	1.501 (7)	C83—H83A	0.9900
C10—C18	1.510 (7)	C83—H83B	0.9900
C10—C11	1.550 (7)	C84—H84A	0.9900
C10—H10	1.0000	C84—H84B	0.9900
C11—C12	1.546 (7)	O85—C86	1.396 (8)
C11—H11A	0.9900	O85—C89	1.398 (8)
C11—H11B	0.9900	C86—C87	1.493 (9)
C12—H12A	0.9900	C86—H86A	0.9900
C12—H12B	0.9900	C86—H86B	0.9900
C17—C18	1.406 (7)	C87—C88	1.476 (8)
C19—C20	1.409 (7)	C87—H87A	0.9900
C21—C22	1.524 (8)	C87—H87B	0.9900
C21—C23	1.531 (7)	C88—C89	1.466 (8)
C21—H21	1.0000	C88—H88A	0.9900
C22—H22A	0.9800	C88—H88B	0.9900
C22—H22B	0.9800	C89—H89A	0.9900
C22—H22C	0.9800	C89—H89B	0.9900
C23—H23A	0.9800	O90—C94	1.368 (9)
C23—H23B	0.9800	O90—C91	1.392 (10)
C23—H23C	0.9800	C91—C92	1.459 (11)
C25—C26	1.524 (7)	C91—H91A	0.9900
C25—C27	1.539 (7)	C91—H91B	0.9900
C25—H25	1.0000	C92—C93	1.488 (9)
C26—H26A	0.9800	C92—H92A	0.9900
C26—H26B	0.9800	C92—H92B	0.9900
C26—H26C	0.9800	C93—C94	1.458 (10)
C27—H27A	0.9800	C93—H93A	0.9900
C27—H27B	0.9800	C93—H93B	0.9900
C27—H27C	0.9800	C94—H94A	0.9900
C31—C32	1.523 (8)	C94—H94B	0.9900
C31—C33	1.541 (7)	O95—C96	1.386 (12)
C31—H31	1.0000	O95—C99	1.395 (12)
C32—H32A	0.9800	C96—C97	1.457 (13)
C32—H32B	0.9800	C96—H96A	0.9900
C32—H32C	0.9800	C96—H96B	0.9900
C33—H33A	0.9800	C97—C98	1.503 (14)
C33—H33B	0.9800	C97—H97A	0.9900
C33—H33C	0.9800	C97—H97B	0.9900
C35—C37	1.517 (7)	C98—C99	1.442 (13)
C35—C36	1.540 (8)	C98—H98A	0.9900
C35—H35	1.0000	C98—H98B	0.9900
C36—H36A	0.9800	C99—H99A	0.9900
C36—H36B	0.9800	C99—H99B	0.9900
C36—H36C	0.9800	O95B—C99B	1.369 (11)
C37—H37A	0.9800	O95B—C96B	1.400 (12)
C37—H37B	0.9800	C96B—C97B	1.462 (13)
C37—H37C	0.9800	C96B—H96C	0.9900
C41—C42	1.513 (7)	C96B—H96D	0.9900
C41—C43	1.523 (7)	C97B—C98B	1.481 (13)
C41—H41	1.0000	C97B—H97C	0.9900
C42—H42A	0.9800	C97B—H97D	0.9900
C42—H42B	0.9800	C98B—C99B	1.469 (13)
C42—H42C	0.9800	C98B—H98C	0.9900
C43—H43A	0.9800	C98B—H98D	0.9900
C43—H43B	0.9800	C99B—H99C	0.9900
C43—H43C	0.9800	C99B—H99D	0.9900
C45—C46	1.526 (8)	O100—C101	1.385 (7)
C45—C47	1.533 (7)	O100—C104	1.386 (7)
C45—H45	1.0000	C101—C102	1.449 (8)
C46—H46A	0.9800	C101—H10A	0.9900
C46—H46B	0.9800	C101—H10B	0.9900
C46—H46C	0.9800	C102—C103	1.463 (9)
C47—H47A	0.9800	C102—H10C	0.9900
C47—H47B	0.9800	C102—H10D	0.9900
C47—H47C	0.9800	C103—C104	1.441 (8)
C51—C53	1.532 (7)	C103—H10E	0.9900
C51—C52	1.532 (7)	C103—H10F	0.9900
C51—H51	1.0000	C104—H10G	0.9900
C52—H52A	0.9800	C104—H10H	0.9900
			
C13—Pd1—P1	92.48 (16)	H47B—C47—H47C	109.5
C13—Pd1—Cl1	83.25 (16)	C53—C51—C52	110.9 (5)
P1—Pd1—Cl1	169.09 (5)	C53—C51—P2	109.8 (4)
C13—Pd1—Cl3	171.59 (16)	C52—C51—P2	110.7 (4)
P1—Pd1—Cl3	95.90 (5)	C53—C51—H51	108.4
Cl1—Pd1—Cl3	88.39 (5)	C52—C51—H51	108.4
C13—Pd1—Pd2	45.84 (15)	P2—C51—H51	108.4
P1—Pd1—Pd2	127.42 (4)	C51—C52—H52A	109.5
Cl1—Pd1—Pd2	55.02 (3)	C51—C52—H52B	109.5
Cl3—Pd1—Pd2	127.57 (3)	H52A—C52—H52B	109.5
C13—Pd2—P2	92.22 (16)	C51—C52—H52C	109.5
C13—Pd2—Cl1	82.90 (16)	H52A—C52—H52C	109.5
P2—Pd2—Cl1	163.70 (6)	H52B—C52—H52C	109.5
C13—Pd2—Cl4	171.82 (16)	C51—C53—H53A	109.5
P2—Pd2—Cl4	95.20 (5)	C51—C53—H53B	109.5
Cl1—Pd2—Cl4	90.85 (5)	H53A—C53—H53B	109.5
C13—Pd2—Pd1	45.73 (16)	C51—C53—H53C	109.5
P2—Pd2—Pd1	130.03 (4)	H53A—C53—H53C	109.5
Cl1—Pd2—Pd1	54.67 (3)	H53B—C53—H53C	109.5
Cl4—Pd2—Pd1	126.13 (4)	C56—C55—C57	111.5 (5)
C14—Pd3—P3	92.96 (16)	C56—C55—P4	110.9 (4)
C14—Pd3—Cl2	83.43 (15)	C57—C55—P4	109.9 (4)
P3—Pd3—Cl2	168.52 (6)	C56—C55—H55	108.2
C14—Pd3—Cl3	171.08 (16)	C57—C55—H55	108.2
P3—Pd3—Cl3	95.94 (5)	P4—C55—H55	108.2
Cl2—Pd3—Cl3	87.73 (5)	C55—C56—H56A	109.5
C14—Pd3—Pd4	45.81 (15)	C55—C56—H56B	109.5
P3—Pd3—Pd4	128.42 (4)	H56A—C56—H56B	109.5
Cl2—Pd3—Pd4	55.00 (3)	C55—C56—H56C	109.5
Cl3—Pd3—Pd4	126.99 (3)	H56A—C56—H56C	109.5
C14—Pd4—P4	93.15 (15)	H56B—C56—H56C	109.5
C14—Pd4—Cl2	82.81 (15)	C55—C57—H57A	109.5
P4—Pd4—Cl2	165.38 (6)	C55—C57—H57B	109.5
C14—Pd4—Cl4	171.78 (15)	H57A—C57—H57B	109.5
P4—Pd4—Cl4	94.75 (5)	C55—C57—H57C	109.5
Cl2—Pd4—Cl4	90.06 (5)	H57A—C57—H57C	109.5
C14—Pd4—Pd3	45.46 (15)	H57B—C57—H57C	109.5
P4—Pd4—Pd3	130.09 (4)	C62—C61—C77	117.8 (5)
Cl2—Pd4—Pd3	54.52 (3)	C62—C61—P3	115.2 (4)
Cl4—Pd4—Pd3	126.60 (4)	C77—C61—P3	126.2 (4)
Pd1—Cl1—Pd2	70.30 (4)	C63—C62—C61	122.9 (5)
Pd3—Cl2—Pd4	70.48 (4)	C63—C62—H62	118.5
Pd1—Cl3—Pd3	105.30 (5)	C61—C62—H62	118.5
Pd2—Cl4—Pd4	106.56 (5)	C64—C63—C62	119.0 (5)
Pd1—C13—Pd2	88.4 (2)	C64—C63—H63	120.5
Pd1—C13—H13A	106 (3)	C62—C63—H63	120.5
Pd2—C13—H13A	108 (3)	C78—C64—C63	119.1 (5)
Pd1—C13—H13B	121 (3)	C78—C64—H64	120.4
Pd2—C13—H13B	121 (3)	C63—C64—H64	120.4
H13A—C13—H13B	110 (4)	C66—C65—C80	118.6 (5)
Pd3—C14—Pd4	88.7 (2)	C66—C65—H65	120.7
Pd3—C14—H14A	120 (3)	C80—C65—H65	120.7
Pd4—C14—H14A	116 (3)	C67—C66—C65	120.6 (5)
Pd3—C14—H14B	105 (3)	C67—C66—H66	119.7
Pd4—C14—H14B	110 (3)	C65—C66—H66	119.7
H14A—C14—H14B	114 (4)	C66—C67—C68	122.0 (5)
C21—P1—C1	101.1 (2)	C66—C67—H67	119.0
C21—P1—C31	105.4 (3)	C68—C67—H67	119.0
C1—P1—C31	105.2 (2)	C79—C68—C67	117.6 (5)
C21—P1—Pd1	110.04 (17)	C79—C68—P4	124.9 (4)
C1—P1—Pd1	126.30 (17)	C67—C68—P4	116.7 (4)
C31—P1—Pd1	107.06 (18)	C79—C69—C77	107.3 (4)
C8—P2—C51	106.0 (2)	C79—C69—C72	107.9 (4)
C8—P2—C41	101.1 (2)	C77—C69—C72	105.3 (4)
C51—P2—C41	103.6 (2)	C79—C69—H69	112.0
C8—P2—Pd2	124.66 (17)	C77—C69—H69	112.0
C51—P2—Pd2	107.31 (18)	C72—C69—H69	112.0
C41—P2—Pd2	112.23 (17)	C80—C70—C78	105.5 (4)
C25—P3—C61	101.7 (2)	C80—C70—C71	108.2 (5)
C25—P3—C35	105.2 (2)	C78—C70—C71	108.1 (4)
C61—P3—C35	104.6 (2)	C80—C70—H70	111.6
C25—P3—Pd3	109.80 (17)	C78—C70—H70	111.6
C61—P3—Pd3	126.43 (16)	C71—C70—H70	111.6
C35—P3—Pd3	107.38 (18)	C70—C71—C72	109.1 (4)
C68—P4—C45	101.5 (2)	C70—C71—H71A	109.9
C68—P4—C55	105.9 (2)	C72—C71—H71A	109.9
C45—P4—C55	104.2 (3)	C70—C71—H71B	109.9
C68—P4—Pd4	124.74 (17)	C72—C71—H71B	109.9
C45—P4—Pd4	111.81 (18)	H71A—C71—H71B	108.3
C55—P4—Pd4	106.91 (18)	C71—C72—C69	110.3 (4)
C2—C1—C17	117.0 (5)	C71—C72—H72A	109.6
C2—C1—P1	115.6 (4)	C69—C72—H72A	109.6
C17—C1—P1	126.8 (4)	C71—C72—H72B	109.6
C3—C2—C1	122.7 (5)	C69—C72—H72B	109.6
C3—C2—H2	118.6	H72A—C72—H72B	108.1
C1—C2—H2	118.6	C78—C77—C61	117.8 (5)
C4—C3—C2	120.5 (5)	C78—C77—C69	111.9 (4)
C4—C3—H3	119.8	C61—C77—C69	130.3 (4)
C2—C3—H3	119.8	C64—C78—C77	123.1 (5)
C3—C4—C18	117.9 (5)	C64—C78—C70	123.4 (5)
C3—C4—H4	121.1	C77—C78—C70	113.4 (5)
C18—C4—H4	121.1	C68—C79—C80	119.4 (5)
C20—C5—C6	118.7 (5)	C68—C79—C69	130.0 (4)
C20—C5—H5	120.6	C80—C79—C69	110.6 (5)
C6—C5—H5	120.6	C65—C80—C79	121.4 (5)
C7—C6—C5	119.4 (5)	C65—C80—C70	123.3 (5)
C7—C6—H6	120.3	C79—C80—C70	115.3 (5)
C5—C6—H6	120.3	C84—O80—C81	107.5 (6)
C6—C7—C8	123.0 (5)	O80—C81—C82	108.9 (6)
C6—C7—H7	118.5	O80—C81—H81A	109.9
C8—C7—H7	118.5	C82—C81—H81A	109.9
C19—C8—C7	117.6 (5)	O80—C81—H81B	109.9
C19—C8—P2	124.7 (4)	C82—C81—H81B	109.9
C7—C8—P2	116.7 (4)	H81A—C81—H81B	108.3
C17—C9—C12	107.1 (4)	C81—C82—C83	101.9 (6)
C17—C9—C19	105.5 (4)	C81—C82—H82A	111.4
C12—C9—C19	107.9 (4)	C83—C82—H82A	111.4
C17—C9—H9	112.0	C81—C82—H82B	111.4
C12—C9—H9	112.0	C83—C82—H82B	111.4
C19—C9—H9	112.0	H82A—C82—H82B	109.2
C20—C10—C18	105.0 (4)	C84—C83—C82	102.3 (6)
C20—C10—C11	108.4 (4)	C84—C83—H83A	111.3
C18—C10—C11	108.5 (4)	C82—C83—H83A	111.3
C20—C10—H10	111.5	C84—C83—H83B	111.3
C18—C10—H10	111.5	C82—C83—H83B	111.3
C11—C10—H10	111.5	H83A—C83—H83B	109.2
C12—C11—C10	109.0 (4)	O80—C84—C83	107.8 (6)
C12—C11—H11A	109.9	O80—C84—H84A	110.1
C10—C11—H11A	109.9	C83—C84—H84A	110.1
C12—C11—H11B	109.9	O80—C84—H84B	110.1
C10—C11—H11B	109.9	C83—C84—H84B	110.1
H11A—C11—H11B	108.3	H84A—C84—H84B	108.5
C9—C12—C11	110.4 (4)	C86—O85—C89	108.4 (6)
C9—C12—H12A	109.6	O85—C86—C87	107.0 (6)
C11—C12—H12A	109.6	O85—C86—H86A	110.3
C9—C12—H12B	109.6	C87—C86—H86A	110.3
C11—C12—H12B	109.6	O85—C86—H86B	110.3
H12A—C12—H12B	108.1	C87—C86—H86B	110.3
C1—C17—C18	119.1 (4)	H86A—C86—H86B	108.6
C1—C17—C9	129.5 (4)	C88—C87—C86	103.1 (6)
C18—C17—C9	111.4 (4)	C88—C87—H87A	111.1
C4—C18—C17	122.6 (5)	C86—C87—H87A	111.1
C4—C18—C10	123.1 (5)	C88—C87—H87B	111.1
C17—C18—C10	114.2 (4)	C86—C87—H87B	111.1
C8—C19—C20	118.7 (5)	H87A—C87—H87B	109.1
C8—C19—C9	129.7 (4)	C89—C88—C87	101.5 (6)
C20—C19—C9	111.6 (4)	C89—C88—H88A	111.5
C5—C20—C19	122.1 (5)	C87—C88—H88A	111.5
C5—C20—C10	123.9 (5)	C89—C88—H88B	111.5
C19—C20—C10	113.9 (5)	C87—C88—H88B	111.5
C22—C21—C23	111.5 (5)	H88A—C88—H88B	109.3
C22—C21—P1	109.1 (4)	O85—C89—C88	105.8 (6)
C23—C21—P1	116.8 (4)	O85—C89—H89A	110.6
C22—C21—H21	106.3	C88—C89—H89A	110.6
C23—C21—H21	106.3	O85—C89—H89B	110.6
P1—C21—H21	106.3	C88—C89—H89B	110.6
C21—C22—H22A	109.5	H89A—C89—H89B	108.7
C21—C22—H22B	109.5	C94—O90—C91	106.6 (8)
H22A—C22—H22B	109.5	O90—C91—C92	111.8 (8)
C21—C22—H22C	109.5	O90—C91—H91A	109.3
H22A—C22—H22C	109.5	C92—C91—H91A	109.3
H22B—C22—H22C	109.5	O90—C91—H91B	109.3
C21—C23—H23A	109.5	C92—C91—H91B	109.3
C21—C23—H23B	109.5	H91A—C91—H91B	107.9
H23A—C23—H23B	109.5	C91—C92—C93	102.1 (7)
C21—C23—H23C	109.5	C91—C92—H92A	111.3
H23A—C23—H23C	109.5	C93—C92—H92A	111.3
H23B—C23—H23C	109.5	C91—C92—H92B	111.3
C26—C25—C27	111.3 (5)	C93—C92—H92B	111.3
C26—C25—P3	110.2 (4)	H92A—C92—H92B	109.2
C27—C25—P3	116.7 (4)	C94—C93—C92	104.3 (7)
C26—C25—H25	106.0	C94—C93—H93A	110.9
C27—C25—H25	106.0	C92—C93—H93A	110.9
P3—C25—H25	106.0	C94—C93—H93B	110.9
C25—C26—H26A	109.5	C92—C93—H93B	110.9
C25—C26—H26B	109.5	H93A—C93—H93B	108.9
H26A—C26—H26B	109.5	O90—C94—C93	110.4 (8)
C25—C26—H26C	109.5	O90—C94—H94A	109.6
H26A—C26—H26C	109.5	C93—C94—H94A	109.6
H26B—C26—H26C	109.5	O90—C94—H94B	109.6
C25—C27—H27A	109.5	C93—C94—H94B	109.6
C25—C27—H27B	109.5	H94A—C94—H94B	108.1
H27A—C27—H27B	109.5	C96—O95—C99	108.5 (11)
C25—C27—H27C	109.5	O95—C96—C97	108.8 (12)
H27A—C27—H27C	109.5	O95—C96—H96A	109.9
H27B—C27—H27C	109.5	C97—C96—H96A	109.9
C32—C31—C33	111.6 (5)	O95—C96—H96B	109.9
C32—C31—P1	110.6 (4)	C97—C96—H96B	109.9
C33—C31—P1	110.1 (4)	H96A—C96—H96B	108.3
C32—C31—H31	108.2	C96—C97—C98	103.2 (12)
C33—C31—H31	108.2	C96—C97—H97A	111.1
P1—C31—H31	108.2	C98—C97—H97A	111.1
C31—C32—H32A	109.5	C96—C97—H97B	111.1
C31—C32—H32B	109.5	C98—C97—H97B	111.1
H32A—C32—H32B	109.5	H97A—C97—H97B	109.1
C31—C32—H32C	109.5	C99—C98—C97	106.3 (11)
H32A—C32—H32C	109.5	C99—C98—H98A	110.5
H32B—C32—H32C	109.5	C97—C98—H98A	110.5
C31—C33—H33A	109.5	C99—C98—H98B	110.5
C31—C33—H33B	109.5	C97—C98—H98B	110.5
H33A—C33—H33B	109.5	H98A—C98—H98B	108.7
C31—C33—H33C	109.5	O95—C99—C98	108.6 (11)
H33A—C33—H33C	109.5	O95—C99—H99A	110.0
H33B—C33—H33C	109.5	C98—C99—H99A	110.0
C37—C35—C36	111.8 (5)	O95—C99—H99B	110.0
C37—C35—P3	110.0 (4)	C98—C99—H99B	110.0
C36—C35—P3	110.2 (4)	H99A—C99—H99B	108.4
C37—C35—H35	108.2	C99B—O95B—C96B	108.7 (10)
C36—C35—H35	108.2	O95B—C96B—C97B	106.9 (10)
P3—C35—H35	108.2	O95B—C96B—H96C	110.3
C35—C36—H36A	109.5	C97B—C96B—H96C	110.3
C35—C36—H36B	109.5	O95B—C96B—H96D	110.3
H36A—C36—H36B	109.5	C97B—C96B—H96D	110.3
C35—C36—H36C	109.5	H96C—C96B—H96D	108.6
H36A—C36—H36C	109.5	C96B—C97B—C98B	105.2 (10)
H36B—C36—H36C	109.5	C96B—C97B—H97C	110.7
C35—C37—H37A	109.5	C98B—C97B—H97C	110.7
C35—C37—H37B	109.5	C96B—C97B—H97D	110.7
H37A—C37—H37B	109.5	C98B—C97B—H97D	110.7
C35—C37—H37C	109.5	H97C—C97B—H97D	108.8
H37A—C37—H37C	109.5	C99B—C98B—C97B	103.5 (10)
H37B—C37—H37C	109.5	C99B—C98B—H98C	111.1
C42—C41—C43	110.8 (5)	C97B—C98B—H98C	111.1
C42—C41—P2	110.6 (4)	C99B—C98B—H98D	111.1
C43—C41—P2	115.4 (4)	C97B—C98B—H98D	111.1
C42—C41—H41	106.5	H98C—C98B—H98D	109.0
C43—C41—H41	106.5	O95B—C99B—C98B	105.4 (11)
P2—C41—H41	106.5	O95B—C99B—H99C	110.7
C41—C42—H42A	109.5	C98B—C99B—H99C	110.7
C41—C42—H42B	109.5	O95B—C99B—H99D	110.7
H42A—C42—H42B	109.5	C98B—C99B—H99D	110.7
C41—C42—H42C	109.5	H99C—C99B—H99D	108.8
H42A—C42—H42C	109.5	C101—O100—C104	109.5 (6)
H42B—C42—H42C	109.5	O100—C101—C102	108.5 (6)
C41—C43—H43A	109.5	O100—C101—H10A	110.0
C41—C43—H43B	109.5	C102—C101—H10A	110.0
H43A—C43—H43B	109.5	O100—C101—H10B	110.0
C41—C43—H43C	109.5	C102—C101—H10B	110.0
H43A—C43—H43C	109.5	H10A—C101—H10B	108.4
H43B—C43—H43C	109.5	C101—C102—C103	106.3 (6)
C46—C45—C47	110.6 (5)	C101—C102—H10C	110.5
C46—C45—P4	110.1 (4)	C103—C102—H10C	110.5
C47—C45—P4	115.7 (4)	C101—C102—H10D	110.5
C46—C45—H45	106.6	C103—C102—H10D	110.5
C47—C45—H45	106.6	H10C—C102—H10D	108.7
P4—C45—H45	106.6	C104—C103—C102	105.8 (6)
C45—C46—H46A	109.5	C104—C103—H10E	110.6
C45—C46—H46B	109.5	C102—C103—H10E	110.6
H46A—C46—H46B	109.5	C104—C103—H10F	110.6
C45—C46—H46C	109.5	C102—C103—H10F	110.6
H46A—C46—H46C	109.5	H10E—C103—H10F	108.7
H46B—C46—H46C	109.5	O100—C104—C103	108.9 (6)
C45—C47—H47A	109.5	O100—C104—H10G	109.9
C45—C47—H47B	109.5	C103—C104—H10G	109.9
H47A—C47—H47B	109.5	O100—C104—H10H	109.9
C45—C47—H47C	109.5	C103—C104—H10H	109.9
H47A—C47—H47C	109.5	H10G—C104—H10H	108.3
			
P1—Pd1—Pd2—C13	−48.2 (2)	C11—C10—C18—C17	−58.7 (6)
Cl1—Pd1—Pd2—C13	118.6 (2)	C7—C8—C19—C20	−7.6 (7)
Cl3—Pd1—Pd2—C13	173.1 (2)	P2—C8—C19—C20	160.4 (4)
C13—Pd1—Pd2—P2	41.6 (2)	C7—C8—C19—C9	172.3 (5)
P1—Pd1—Pd2—P2	−6.58 (8)	P2—C8—C19—C9	−19.7 (8)
Cl1—Pd1—Pd2—P2	160.23 (7)	C17—C9—C19—C8	−124.8 (5)
Cl3—Pd1—Pd2—P2	−145.33 (7)	C12—C9—C19—C8	120.9 (5)
C13—Pd1—Pd2—Cl1	−118.6 (2)	C17—C9—C19—C20	55.1 (5)
P1—Pd1—Pd2—Cl1	−166.80 (7)	C12—C9—C19—C20	−59.2 (5)
Cl3—Pd1—Pd2—Cl1	54.44 (6)	C6—C5—C20—C19	1.6 (8)
C13—Pd1—Pd2—Cl4	−179.0 (2)	C6—C5—C20—C10	−175.2 (5)
P1—Pd1—Pd2—Cl4	132.84 (7)	C8—C19—C20—C5	4.9 (8)
Cl1—Pd1—Pd2—Cl4	−60.35 (7)	C9—C19—C20—C5	−175.0 (5)
Cl3—Pd1—Pd2—Cl4	−5.91 (7)	C8—C19—C20—C10	−178.0 (4)
P3—Pd3—Pd4—C14	−47.2 (2)	C9—C19—C20—C10	2.1 (6)
Cl2—Pd3—Pd4—C14	119.1 (2)	C18—C10—C20—C5	118.9 (6)
Cl3—Pd3—Pd4—C14	173.1 (2)	C11—C10—C20—C5	−125.3 (6)
C14—Pd3—Pd4—P4	43.3 (2)	C18—C10—C20—C19	−58.1 (6)
P3—Pd3—Pd4—P4	−3.91 (8)	C11—C10—C20—C19	57.7 (6)
Cl2—Pd3—Pd4—P4	162.40 (7)	C1—P1—C21—C22	−66.9 (4)
Cl3—Pd3—Pd4—P4	−143.62 (7)	C31—P1—C21—C22	−176.2 (4)
C14—Pd3—Pd4—Cl2	−119.1 (2)	Pd1—P1—C21—C22	68.7 (4)
P3—Pd3—Pd4—Cl2	−166.31 (7)	C1—P1—C21—C23	60.6 (5)
Cl3—Pd3—Pd4—Cl2	53.98 (7)	C31—P1—C21—C23	−48.7 (5)
C14—Pd3—Pd4—Cl4	−177.2 (2)	Pd1—P1—C21—C23	−163.8 (4)
P3—Pd3—Pd4—Cl4	135.55 (7)	C61—P3—C25—C26	−67.4 (4)
Cl2—Pd3—Pd4—Cl4	−58.15 (8)	C35—P3—C25—C26	−176.2 (4)
Cl3—Pd3—Pd4—Cl4	−4.16 (8)	Pd3—P3—C25—C26	68.6 (4)
C13—Pd1—Cl1—Pd2	39.34 (16)	C61—P3—C25—C27	60.7 (4)
P1—Pd1—Cl1—Pd2	106.8 (3)	C35—P3—C25—C27	−48.1 (5)
Cl3—Pd1—Cl1—Pd2	−139.83 (5)	Pd3—P3—C25—C27	−163.3 (4)
C13—Pd2—Cl1—Pd1	−39.29 (16)	C21—P1—C31—C32	175.1 (4)
P2—Pd2—Cl1—Pd1	−112.65 (17)	C1—P1—C31—C32	68.7 (4)
Cl4—Pd2—Cl1—Pd1	135.41 (5)	Pd1—P1—C31—C32	−67.8 (4)
C14—Pd3—Cl2—Pd4	39.11 (16)	C21—P1—C31—C33	−61.2 (4)
P3—Pd3—Cl2—Pd4	111.3 (2)	C1—P1—C31—C33	−167.5 (4)
Cl3—Pd3—Cl2—Pd4	−139.72 (5)	Pd1—P1—C31—C33	56.0 (4)
C14—Pd4—Cl2—Pd3	−38.89 (15)	C25—P3—C35—C37	−65.2 (5)
P4—Pd4—Cl2—Pd3	−113.6 (2)	C61—P3—C35—C37	−171.9 (4)
Cl4—Pd4—Cl2—Pd3	137.01 (5)	Pd3—P3—C35—C37	51.7 (5)
C13—Pd1—Cl3—Pd3	33.1 (12)	C25—P3—C35—C36	171.1 (4)
P1—Pd1—Cl3—Pd3	−151.36 (6)	C61—P3—C35—C36	64.4 (4)
Cl1—Pd1—Cl3—Pd3	38.70 (6)	Pd3—P3—C35—C36	−72.0 (4)
Pd2—Pd1—Cl3—Pd3	−3.13 (7)	C8—P2—C41—C42	67.7 (4)
C14—Pd3—Cl3—Pd1	41.5 (10)	C51—P2—C41—C42	177.3 (4)
P3—Pd3—Cl3—Pd1	−141.93 (6)	Pd2—P2—C41—C42	−67.2 (4)
Cl2—Pd3—Cl3—Pd1	48.98 (6)	C8—P2—C41—C43	−59.1 (5)
Pd4—Pd3—Cl3—Pd1	7.45 (7)	C51—P2—C41—C43	50.6 (5)
C13—Pd2—Cl4—Pd4	3.9 (12)	Pd2—P2—C41—C43	166.0 (4)
P2—Pd2—Cl4—Pd4	158.94 (7)	C68—P4—C45—C46	68.6 (4)
Cl1—Pd2—Cl4—Pd4	−36.21 (7)	C55—P4—C45—C46	178.4 (4)
Pd1—Pd2—Cl4—Pd4	8.95 (9)	Pd4—P4—C45—C46	−66.4 (4)
C14—Pd4—Cl4—Pd2	−18.7 (11)	C68—P4—C45—C47	−57.7 (5)
P4—Pd4—Cl4—Pd2	145.37 (7)	C55—P4—C45—C47	52.2 (5)
Cl2—Pd4—Cl4—Pd2	−48.46 (7)	Pd4—P4—C45—C47	167.3 (4)
Pd3—Pd4—Cl4—Pd2	−4.70 (9)	C8—P2—C51—C53	172.1 (4)
P1—Pd1—C13—Pd2	143.68 (16)	C41—P2—C51—C53	66.1 (4)
Cl1—Pd1—C13—Pd2	−46.40 (15)	Pd2—P2—C51—C53	−52.8 (4)
Cl3—Pd1—C13—Pd2	−40.7 (13)	C8—P2—C51—C52	−65.1 (4)
P2—Pd2—C13—Pd1	−149.43 (16)	C41—P2—C51—C52	−171.1 (4)
Cl1—Pd2—C13—Pd1	46.18 (15)	Pd2—P2—C51—C52	70.0 (4)
Cl4—Pd2—C13—Pd1	5.7 (13)	C68—P4—C55—C56	−67.6 (4)
P3—Pd3—C14—Pd4	144.83 (15)	C45—P4—C55—C56	−174.2 (4)
Cl2—Pd3—C14—Pd4	−46.11 (14)	Pd4—P4—C55—C56	67.2 (4)
Cl3—Pd3—C14—Pd4	−38.6 (11)	C68—P4—C55—C57	168.6 (4)
P4—Pd4—C14—Pd3	−148.28 (15)	C45—P4—C55—C57	62.1 (5)
Cl2—Pd4—C14—Pd3	45.84 (14)	Pd4—P4—C55—C57	−56.5 (4)
Cl4—Pd4—C14—Pd3	15.8 (12)	C25—P3—C61—C62	−73.1 (4)
C13—Pd1—P1—C21	−158.8 (3)	C35—P3—C61—C62	36.1 (5)
Cl1—Pd1—P1—C21	134.6 (3)	Pd3—P3—C61—C62	161.2 (3)
Cl3—Pd1—P1—C21	21.9 (2)	C25—P3—C61—C77	96.6 (5)
Pd2—Pd1—P1—C21	−126.45 (19)	C35—P3—C61—C77	−154.1 (4)
C13—Pd1—P1—C1	−37.3 (3)	Pd3—P3—C61—C77	−29.0 (5)
Cl1—Pd1—P1—C1	−103.9 (3)	C77—C61—C62—C63	−2.5 (8)
Cl3—Pd1—P1—C1	143.3 (2)	P3—C61—C62—C63	168.2 (5)
Pd2—Pd1—P1—C1	−5.0 (2)	C61—C62—C63—C64	−0.5 (9)
C13—Pd1—P1—C31	87.2 (2)	C62—C63—C64—C78	2.2 (8)
Cl1—Pd1—P1—C31	20.6 (3)	C80—C65—C66—C67	−4.3 (9)
Cl3—Pd1—P1—C31	−92.19 (19)	C65—C66—C67—C68	2.3 (9)
Pd2—Pd1—P1—C31	119.51 (19)	C66—C67—C68—C79	3.4 (8)
C13—Pd2—P2—C8	37.3 (3)	C66—C67—C68—P4	−166.7 (5)
Cl1—Pd2—P2—C8	109.4 (3)	C45—P4—C68—C79	−90.5 (5)
Cl4—Pd2—P2—C8	−139.2 (2)	C55—P4—C68—C79	160.9 (4)
Pd1—Pd2—P2—C8	8.9 (2)	Pd4—P4—C68—C79	36.5 (5)
C13—Pd2—P2—C51	−87.2 (2)	C45—P4—C68—C67	78.7 (4)
Cl1—Pd2—P2—C51	−15.1 (3)	C55—P4—C68—C67	−29.8 (5)
Cl4—Pd2—P2—C51	96.27 (18)	Pd4—P4—C68—C67	−154.2 (3)
Pd1—Pd2—P2—C51	−115.58 (18)	C80—C70—C71—C72	−58.2 (6)
C13—Pd2—P2—C41	159.7 (2)	C78—C70—C71—C72	55.6 (6)
Cl1—Pd2—P2—C41	−128.2 (2)	C70—C71—C72—C69	3.4 (6)
Cl4—Pd2—P2—C41	−16.86 (19)	C79—C69—C72—C71	54.6 (6)
Pd1—Pd2—P2—C41	131.30 (19)	C77—C69—C72—C71	−59.8 (5)
C14—Pd3—P3—C25	−158.6 (2)	C62—C61—C77—C78	3.6 (7)
Cl2—Pd3—P3—C25	130.1 (3)	P3—C61—C77—C78	−165.9 (4)
Cl3—Pd3—P3—C25	21.90 (18)	C62—C61—C77—C69	−176.4 (5)
Pd4—Pd3—P3—C25	−126.81 (18)	P3—C61—C77—C69	14.1 (8)
C14—Pd3—P3—C61	−36.4 (3)	C79—C69—C77—C78	−55.4 (5)
Cl2—Pd3—P3—C61	−107.7 (3)	C72—C69—C77—C78	59.4 (5)
Cl3—Pd3—P3—C61	144.1 (2)	C79—C69—C77—C61	124.6 (5)
Pd4—Pd3—P3—C61	−4.6 (2)	C72—C69—C77—C61	−120.6 (5)
C14—Pd3—P3—C35	87.5 (2)	C63—C64—C78—C77	−1.0 (8)
Cl2—Pd3—P3—C35	16.2 (3)	C63—C64—C78—C70	177.0 (5)
Cl3—Pd3—P3—C35	−91.94 (19)	C61—C77—C78—C64	−2.0 (8)
Pd4—Pd3—P3—C35	119.34 (18)	C69—C77—C78—C64	178.0 (5)
C14—Pd4—P4—C68	34.1 (3)	C61—C77—C78—C70	179.8 (4)
Cl2—Pd4—P4—C68	107.5 (3)	C69—C77—C78—C70	−0.2 (6)
Cl4—Pd4—P4—C68	−143.6 (2)	C80—C70—C78—C64	−122.2 (6)
Pd3—Pd4—P4—C68	4.8 (2)	C71—C70—C78—C64	122.2 (6)
C14—Pd4—P4—C45	156.7 (3)	C80—C70—C78—C77	56.0 (6)
Cl2—Pd4—P4—C45	−129.9 (3)	C71—C70—C78—C77	−59.6 (6)
Cl4—Pd4—P4—C45	−21.0 (2)	C67—C68—C79—C80	−6.9 (7)
Pd3—Pd4—P4—C45	127.4 (2)	P4—C68—C79—C80	162.2 (4)
C14—Pd4—P4—C55	−89.8 (2)	C67—C68—C79—C69	172.2 (5)
Cl2—Pd4—P4—C55	−16.4 (3)	P4—C68—C79—C69	−18.6 (8)
Cl4—Pd4—P4—C55	92.41 (19)	C77—C69—C79—C68	−126.1 (6)
Pd3—Pd4—P4—C55	−119.16 (19)	C72—C69—C79—C68	120.9 (6)
C21—P1—C1—C2	−75.0 (4)	C77—C69—C79—C80	53.1 (5)
C31—P1—C1—C2	34.5 (5)	C72—C69—C79—C80	−59.9 (6)
Pd1—P1—C1—C2	159.7 (3)	C66—C65—C80—C79	0.5 (9)
C21—P1—C1—C17	95.5 (5)	C66—C65—C80—C70	−177.1 (5)
C31—P1—C1—C17	−155.0 (5)	C68—C79—C80—C65	5.2 (8)
Pd1—P1—C1—C17	−29.8 (5)	C69—C79—C80—C65	−174.2 (5)
C17—C1—C2—C3	−4.0 (8)	C68—C79—C80—C70	−177.0 (5)
P1—C1—C2—C3	167.4 (4)	C69—C79—C80—C70	3.6 (7)
C1—C2—C3—C4	−0.5 (9)	C78—C70—C80—C65	119.0 (6)
C2—C3—C4—C18	3.5 (8)	C71—C70—C80—C65	−125.5 (6)
C20—C5—C6—C7	−5.1 (8)	C78—C70—C80—C79	−58.8 (6)
C5—C6—C7—C8	2.2 (9)	C71—C70—C80—C79	56.7 (6)
C6—C7—C8—C19	4.2 (8)	C84—O80—C81—C82	5.0 (9)
C6—C7—C8—P2	−164.8 (4)	O80—C81—C82—C83	−24.0 (9)
C51—P2—C8—C19	160.6 (4)	C81—C82—C83—C84	32.6 (8)
C41—P2—C8—C19	−91.7 (5)	C81—O80—C84—C83	16.9 (9)
Pd2—P2—C8—C19	35.5 (5)	C82—C83—C84—O80	−31.4 (8)
C51—P2—C8—C7	−31.3 (5)	C89—O85—C86—C87	−4.0 (12)
C41—P2—C8—C7	76.4 (4)	O85—C86—C87—C88	−19.3 (11)
Pd2—P2—C8—C7	−156.4 (3)	C86—C87—C88—C89	33.6 (10)
C20—C10—C11—C12	−58.4 (6)	C86—O85—C89—C88	26.2 (11)
C18—C10—C11—C12	55.2 (6)	C87—C88—C89—O85	−37.3 (9)
C17—C9—C12—C11	−58.4 (5)	C94—O90—C91—C92	2.7 (16)
C19—C9—C12—C11	54.8 (5)	O90—C91—C92—C93	−15.2 (15)
C10—C11—C12—C9	2.3 (6)	C91—C92—C93—C94	20.8 (12)
C2—C1—C17—C18	5.4 (7)	C91—O90—C94—C93	11.8 (14)
P1—C1—C17—C18	−165.0 (4)	C92—C93—C94—O90	−21.1 (13)
C2—C1—C17—C9	−174.2 (5)	C99—O95—C96—C97	22 (4)
P1—C1—C17—C9	15.4 (8)	O95—C96—C97—C98	−20 (4)
C12—C9—C17—C1	−121.6 (6)	C96—C97—C98—C99	11 (4)
C19—C9—C17—C1	123.6 (5)	C96—O95—C99—C98	−15 (3)
C12—C9—C17—C18	58.7 (5)	C97—C98—C99—O95	2(4)
C19—C9—C17—C18	−56.0 (5)	C99B—O95B—C96B—C97B	−21 (3)
C3—C4—C18—C17	−2.0 (8)	O95B—C96B—C97B—C98B	1(3)
C3—C4—C18—C10	174.8 (5)	C96B—C97B—C98B—C99B	17 (3)
C1—C17—C18—C4	−2.5 (8)	C96B—O95B—C99B—C98B	32 (2)
C9—C17—C18—C4	177.2 (5)	C97B—C98B—C99B—O95B	−30 (3)
C1—C17—C18—C10	−179.6 (4)	C104—O100—C101—C102	−7.5 (10)
C9—C17—C18—C10	0.1 (6)	O100—C101—C102—C103	1.9 (11)
C20—C10—C18—C4	−120.0 (5)	C101—C102—C103—C104	4.1 (12)
C11—C10—C18—C4	124.3 (5)	C101—O100—C104—C103	10.3 (11)
C20—C10—C18—C17	57.1 (6)	C102—C103—C104—O100	−8.7 (12)
